# Cross-lagged analysis of mobile phone addiction and bedtime procrastination: a comparison of gender and perceived stress levels among Chinese college students

**DOI:** 10.3389/fpsyg.2025.1588090

**Published:** 2025-05-14

**Authors:** Xiujian Lin, Xueping Fu, Yuhao Shen, Gaoyang Liu, Ningning Ding, Guohua Zhang, Jun Qian

**Affiliations:** ^1^School of Mental Health, Wenzhou Medical University, Wenzhou, China; ^2^Renji College, Wenzhou Medical University, Wenzhou, China

**Keywords:** bedtime procrastination, perceived stress levels, gender difference, mobile phone addiction, college students

## Abstract

**Purpose:**

This study aims to investigate the longitudinal relationship between Mobile Phone Addiction (MPA) and Bedtime Procrastination (BP) and to analyze differences across various gender and stress level groups, providing a theoretical basis and practical guidance for the development of relevant intervention strategies.

**Methods:**

A longitudinal research design was employed, conducting two surveys among 1,423 first-year to junior college students at a university in Wenzhou, Zhejiang Province, in November 2021 and May 2022. Measurements were taken using the Mobile Phone Addiction Scale, Bedtime Procrastination Scale, and Depression Anxiety Stress Scale-21 (DASS-21). Data were analyzed using SPSS 26.0 and AMOS 24.0, employing methods such as descriptive statistics, correlation analysis, repeated measures variance analysis, and structural equation modeling.

**Results:**

The study found that both MPA and BP are on the rise among college students and share a bidirectional longitudinal relationship. Under low-stress conditions, MPA significantly predicted subsequent BP, while under high-stress, BP significantly predicted subsequent MPA. Additionally, gender-specific analyses revealed that MPA significantly predicted subsequent BP in both male and female students, but BP only significantly predicted subsequent MPA in female students.

**Conclusion:**

MPA and BP mutually influence each other among college students, with this relationship being moderated by stress levels and gender. Personalized intervention measures should be adopted for college students of different genders and stress levels to effectively prevent and alleviate MPA and BP, promoting the healthy growth and comprehensive development of college students.

## 1 Introduction

Technological advancements have made mobile phones essential for modern communication. As of June 2024, China boasts a mobile internet user base of 1.09 billion, with 99.7% of internet users accessing the web via mobile devices. Among them, 13.5% are college students aged 20–29 (China Internet Network Information Center, 2024). While mobile phones facilitate communication and daily tasks, their overuse has led to several issues that particularly affect the physical and mental health of young people, such as anxiety and depression (Samaha and Hawi, 2016; Enez Darcin et al., 2016; Zhang et al., 2019). Furthermore, the excessive use of mobile phones might also lead to Mobile Phone Addiction (MPA), which refers to cognitive, emotional, and behavioral changes resulting from excessive usage and is marked by emotional dependence despite an awareness of its negative effects (Winkler et al., 2020).

MPA is prevalent among college students. In China, 40% of students feel uneasy without their phones, while 37% struggle when separated from their devices (Zhu et al., 2009). A meta-analysis reveals that 23% of Chinese college students suffer from MPA, with contributing factors including abundant free time, low self-control, and increased pressures related to identity and lifestyle demands, such as online learning, socializing, gaming, and shopping (Winkler et al., 2020). Recent studies have found that MPA might negatively impact academic performance (Hong et al., 2021), social interactions (Yusuf et al., 2020), and overall well-being (Li et al., 2020). While MPA leads to several health issues, one of the most noticeable detriments might be Bedtime Procrastination (BP), which refers to the intentional delay of bedtime despite the absence of external disruptions (Geng et al., 2021; Meng et al., 2024; Cemei et al., 2024; Bozkurt et al., 2024; Kroese et al., 2014). BP is linked to sleep deprivation (Meng et al., 2024), which in turn is associated with mood disorders, cognitive decline, weakened immunity, and obesity (Ma et al., 2022). Moreover, it might diminish productivity and academic performance, adversely affecting the mental and physical health of college students (Cui et al., 2021).

The relationship between MPA and BP appears to be robust, with research indicating that excessive night-time mobile phone use, driven by addiction, unfavorably impacts sleep quality and duration (Hamvai et al., 2023). Prolonged phone use can decrease melatonin levels as the screen’s blue light inhibits the production of melatonin and makes it harder for the mobile phone users to fall asleep (Vollmer et al., 2017). Meanwhile, BP can also exacerbate these issues by reinforcing addictive behaviors (Yang J. et al., 2020). Activities such as watching videos, reading online novels, or gaming can create an immersive “flow” state, blurring the sense of time and delaying bedtime (Geng et al., 2018; Zeng, 2019; Chung et al., 2020). This not only diminishes sleep quality but can also result in a negative mental state the following day, which impacts learning and daily functioning (Schmidt et al., 2024).

A longitudinal study of 622 Chinese college students found a strong positive correlation between MPA and BP, revealing a bidirectional relationship where MPA increases the risk of BP, and BP contributes to the development of MPA (Chen et al., 2023). Specially, students with higher levels of MPA early on were more likely to develop severe BP later, while those with lower MPA experienced fewer BP issues. This study also emphasized the mediating role of self-control, suggesting that interventions aimed at enhancing self-regulation skills could help alleviate both MPA and BP (Chen et al., 2023). These findings underscore the intricate interplay between cognitive and emotional processes, suggesting that interventions targeting either MPA or BP should consider both individual predispositions and situational factors to address these issues effectively.

To better understand the bidirectional relationship between MPA and BP, we draw on several theoretical frameworks. The Interaction of Person-Affect-Cognition-Execution (I-PACE) model (Brand et al., 2016, 2019) posits that individual predispositions, affective responses, cognitive processes, and executive functions interact to influence addictive behaviors. In the context of MPA and BP, the disparities in self-regulation capacity, emotional regulation, and cognitive biases play a crucial role in the development and persistence of these behaviors. Besides, the Behavioral Addiction Theory (Kuss and Griffiths, 2012) highlights the reinforcing mechanisms underlying addictive behaviors, suggesting that the pleasurable experiences associated with mobile phone use create a reward loop that perpetuates addiction. Furthermore, the Self-Determination Theory (Ryan and Deci, 2000) underscores the importance of autonomy, competence, and relatedness in maintaining healthy behaviors. When it comes to MPA and BP, these needs play a critical role in problematic behaviors. Insufficient autonomy over phone use and the desire for relatedness to sustain peer relationships induce students to excessive mobile phone use, which in turn disrupts sleep routines and contributes to BP (Gao et al., 2022; Hong et al., 2020, 2021).

Given that stress is common among college students and is likely to negatively impact academic performance as well as mental and physical health (Schmidt et al., 2024), it is essential to investigate its role in examining the relationship between MPA and BP. However, empirical research on the daily dynamics of these three constructs among college students remains limited. One recent study in Korea conducted by Song and Park (2019) revealed that stress was positively correlated with internet addiction. Under stress, individuals often use their mobile phones to escape real-life problems and relieve negative emotions (Song and Park, 2019). Furthermore, Schmidt et al. (2024) conducted an investigation on 96 German college students to explore the relationship between stress and BP using a sleep-tracking wearable over 2 weeks. The findings demonstrated that students who experienced more stress on a daily basis had increased BP, shorter sleep time, and lower sleep quality (Schmidt et al., 2024). These findings are consistent with those of Berset et al. (2011), who investigated 393 adults and found that stress was a strong predictor of impaired sleep. Stress might lead to thought backtracking before bedtime, which is likely to disrupt sleep and might contribute to BP (Berset et al., 2011). Meanwhile, Bernecker and Job (2020) found that following a particularly stressful day, individuals appear to be more inclined to procrastinate on sleep. In these contexts, people are more likely to turn to their phones to alleviate stress, which might subsequently lead to bedtime procrastination. This pattern creates a vicious cycle: Stress increases mobile phone use, which in turn worsens BP, perpetuates stress, and further disrupts sleep quality and overall health.

In addition, our study recognizes that gender differences play a significant role in the relationship between MPA and BP. Previous research has highlighted that women tend to exhibit higher levels of MPA than men (Li et al., 2014; Van Deursen et al., 2015). This disparity may be attributed to distinct coping mechanisms for stress, with women typically relying on cognitive reappraisal to manage negative emotions, whereas men are more likely to engage in expressive inhibition (Yang X. et al., 2020). These divergent coping strategies may influence the tendency to use mobile phones as a means of escaping reality during stressful times, thereby shaping the interplay between MPA and BP.

**Figure 1:**
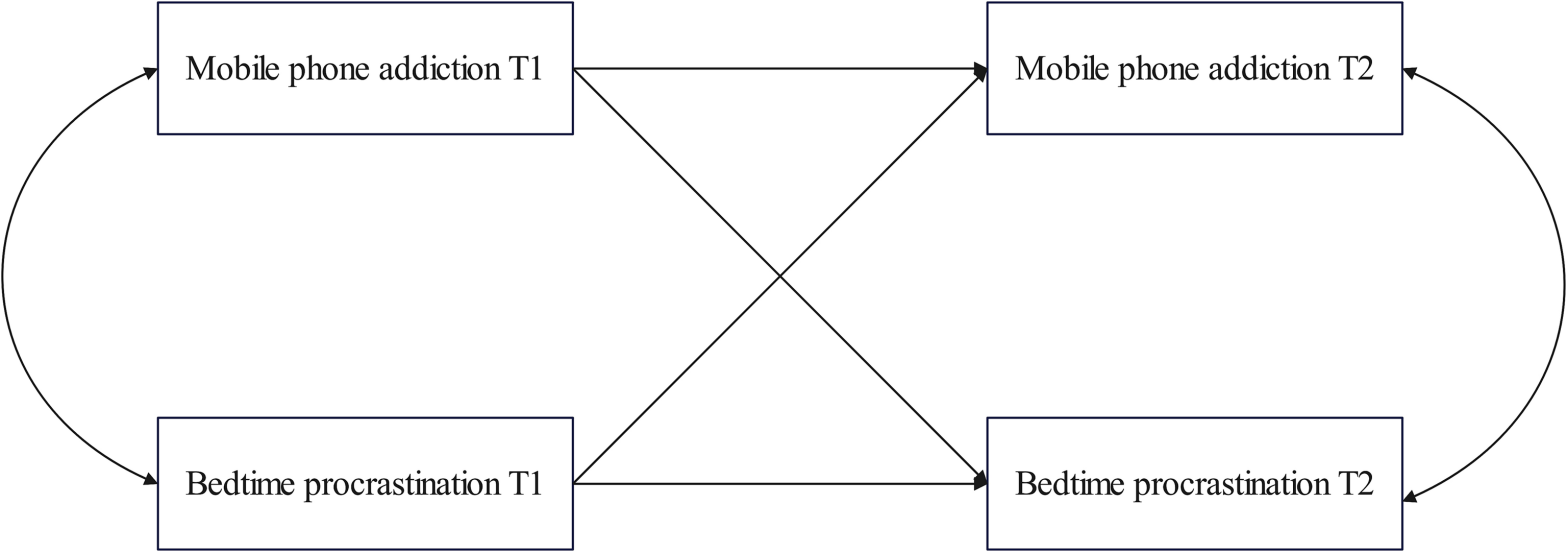
The cross-lagged model of MPA and BP.

Given these theoretical and practical underpinnings, we hypothesize a bidirectional relationship between MPA and BP. Specifically, we propose that MPA at Time 1 (T1) would predict BP at Time 2 (T2), and BP at T1 would predict MPA at T2. This relationship would be moderated by perceived stress levels, with high stress exacerbating the relationship between MPA and BP. Additionally, gender would moderate this relationship, with the relationship being stronger among female students compared to male students. The specific research assumptions are outlined as follows (see Figure 1).

1 There is a bidirectional relationship between MPA and BP among college students over time.
2 Under high-stress conditions, the cross-lagged relationship between MPA and BP is more pronounced among college students than under low-stress conditions.
3 Among female college students, the cross-lagged relationship between MPA and BP is stronger and more closely linked than among male students.

## 2 Methods

### 2.1 Participants and procedures

The study was conducted with a sample of college students from a university in Wenzhou, Zhejiang Province, using the convenience sampling method in November 2021 (T1). Data collection was conducted online via the Wenjuanxing platform, where participants completed the questionnaire anonymously after providing informed consent. The first page of the questionnaire provided comprehensive information about the study’s purpose, significance, completion instructions, and contact details for any questions participants might have. Informed consent was obtained from all participants prior to their participation. Participants completed the questionnaire anonymously after providing their informed consent. To ensure the integrity of the data, participants were required to complete all the questionnaire items before submission. The follow-up survey (T2) was conducted 6 months later in May 2022, with participants identified by their student IDs from the first survey to maintain data consistency.

Participants were invited to take part in the study on a voluntary basis and were not compensated for their participation. The questionnaire was designed to be completed in approximately 15 min. The study adhered to the Declaration of Helsinki and received ethical approval from the ethics committee of the corresponding author’s university (Number: 2022–028). The final dataset included 1,593 college students who completed both rounds of surveys. However, 170 questionnaires were deemed invalid due to reasons such as repeated submissions, lack of serious responses (e.g., patterned answers, incorrect basic information), or exceptionally short response times (less than 120 s). After excluding these invalid responses, 1,423 valid questionnaires were obtained, resulting in an effective recovery rate of 89.3%.

### 2.2 Measure

#### 2.2.1 Background variables

The study collected demographic information about the participants, including age, gender, grade, whether they were an only child, and hometown (urban or rural). Additionally, participants reported their average daily mobile phone use before sleep and the activities they engaged in before sleep at both T1 and T2. The specific categories and frequencies were presented in Table 1.

**Table 1:** Demographic characteristics of the participants.

Variable	*N*	%	Variable	*N*	%
Gender			Average daily mobile phone use before sleep at T1		
Male	276	19.4	< 10 min	21	1.5
Female	1,147	80.6	10–30 min	121	8.5
Only Child			30–60 min	416	29.2
Yes	531	37.3	60–120 min	487	34.2
No	892	62.7	> 120 min	378	26.6
Grade			Average daily mobile phone use before sleep at T2		
Freshman	810	59.6	< 10 min	19	1.3
Sophomore	597	42.0	10–30 min	75	5.3
Junior	16	1.1	30–60 min	355	24.9
Hometown			60–120 min	524	36.8
Urban	572	40.2	> 120 min	450	31.6
Rural	851	59.8	Activities before sleep at T2		
Activities before sleep at T1			Making Calls	36	2.5
Making Calls	33	2.3	Watching Short Videos	179	12.6
Watching Short Videos	165	11.6	Watching TV	67	4.7
Watching TV	54	3.8	Listening to Music	123	8.6
Listening to Music	133	9.3	WeChat/QQ Chatting	274	19.3
WeChat/QQ Chatting	332	23.3	Reading E-books	121	8.5
Reading E-books	118	8.3	Online Shopping	60	4.2
Online Shopping	66	4.6	Browsing News	36	2.5
Browsing News	23	1.6	Online Learning	39	2.7
Online Learning	62	4.4	Playing Games	62	4.4
Playing Games	65	4.6			

#### 2.2.2 Mobile phone addiction

The Mobile Phone Addiction Index (MPAI), developed by Leung (2008), measured problematic mobile phone use among college students and consisted of 17 items across four dimensions: avoidance, loss of control, inefficiency, and withdrawal. Responses were scored on a 5-point Likert scale ranging from 1 (never) to 5 (always), with higher scores indicating higher levels of problematic phone use. The Cronbach’s *α* was 0.91 at T1 and 0.93 at T2, respectively.

#### 2.2.3 Bedtime procrastination

The sleep procrastination scale, developed by Kroese et al. (2014) and adapted by Xu (2017), measured college students’ BP. It consisted of 8 items scored on a 5-point Likert scale, with higher scores indicating a greater tendency to delay sleep. The Cronbach’s α was 0.92 at T1 and 0.93 at T2, respectively.

#### 2.2.4 Perceived stress level

The Depression Anxiety Stress Scale-21 (DASS-21), compiled by Lovibond and Lovibond (1995) and adapted by Gong et al. (2010), was used to assess common emotional disorders such as depression, anxiety, and stress, providing auxiliary psychometric indicators for clinical diagnosis. The scale consisted of three subscales: depression, anxiety, and stress, each containing 7 items. Responses were scored on a 4-point Likert scale, with higher scores indicating higher levels of depression, anxiety, or stress. In this study, only the stress subscale of the DASS-21 was employed at T1, with Cronbach’s α values of 0.91. To differentiate the stress levels, we used the median score of the stress subscale as the cutoff point. Participants scored above the median were classified as the high-stress group, while those scored below the median were classified as the low-stress group.

### 2.3 Statistical analysis

Data were inputted and screened using IBM SPSS Statistics 26.0 (IBM Corporation, Armonk, NY, United States). The analyses included a common method bias test, reliability analysis, descriptive statistics, correlation analysis, difference tests for demographic variables, and repeated measures variance analysis. To examine the bidirectional relationship between MPA and BP, we constructed a cross-lagged model using AMOS 24.0 (also by IBM Corporation). Model fit was evaluated using various indices in AMOS, such as the Comparative Fit Index (CFI), Tucker-Lewis Index (TLI), Root Mean Square Error of Approximation (RMSEA), and Standardized Root Mean Square Residual (SRMR), along with their 90% confidence intervals. An acceptable model fit was determined by CFI and TLI values greater than or equal to 0.90, and RMSEA and SRMR values less than or equal to 0.08.

## 3 Results

### 3.1 Common method bias testing

Given that the data in this study were derived from participant self-reports, common method bias might be present. To mitigate this, the anonymity and confidentiality of the questionnaire were emphasized during the survey administration among college students, and it was clarified that the data would be used solely for scientific research purposes. Additionally, Harman’s single-factor test was employed to assess common method bias (Podsakoff et al., 2003). The results indicated that, without rotation, six factors with eigenvalues greater than 1 were obtained for both the pre-test and post-test. The variance explained by the first factor was 31.47% for the pre-test and 32.74% for the post-test, both of which were below the critical threshold of 40% (Podsakoff et al., 2003). Therefore, it was concluded that there was no significant common method bias in the current study.

### 3.2 Preliminary analyses

#### 3.2.1 Developmental trends of MPA and BP in college students

For MPA, a significant main effect of time was found, *F* (1,1,421) = 4.018, *p* = 0.045, η^2^p = 0.003. Specifically, higher levels of addiction were observed at T2 (M = 54.10, SD = 11.06) compared to T1 (M = 53.46, SD = 10.76). The main effect of gender was not significant and the interaction between time and gender was also not significant. For BP, the main effect of time was significant, F (1,1,421) = 11.34, *p* = 0.001, η^2^p = 0.008, with higher levels of BP at T2 (M = 26.46, SD = 5.56) compared to T1 (M = 25.93, SD = 5.72). The main effect of gender was also significant, F (1,1,421) = 3.966, *p* = 0.047, η^2^p = 0.003, but the interaction between time and gender was not significant. These results indicate that both MPA and BP showed significant changes over time, with BP also being influenced by gender. However, gender differences did not interact with the changes observed over time for either variable.

### 3.3 Correlation analyses

The correlation analysis showed (see Table 2) that there was a significant correlation between MPA and BP over time, indicating that MPA and BP among college students exhibited a certain stability from T1 to T2. In addition, there were significant simultaneous and sequential correlations between MPA and BP at T1 and T2. The above results showed that the synchronization correlation and stability of the variables were consistent, and the correlation conformed to the assumptions of the cross-lagged design.

**Table 2:** Analysis of the correlation between MPA and BP in college students.

Variable	M ± SD	1	2	3	4
1.Mobile Addiction T1	53.46 ± 10.757	1			
2.Mobile Addiction T2	54.10 ± 11.059	0.466^***^			
3.BP T1	25.93 ± 5.719	0.571^***^	0.335^***^		
4.BP T2	26.46 ± 5.560	0.354^***^	0.674^***^	0.446^***^	
5.Stress T1	11.77 ± 4.421	0.492^***^	0.276^***^	0.389^***^	0.217^***^

***p < 0.001.

### 3.4 Cross-lagged analysis of MPA and BP among college students

**Figure 2:**
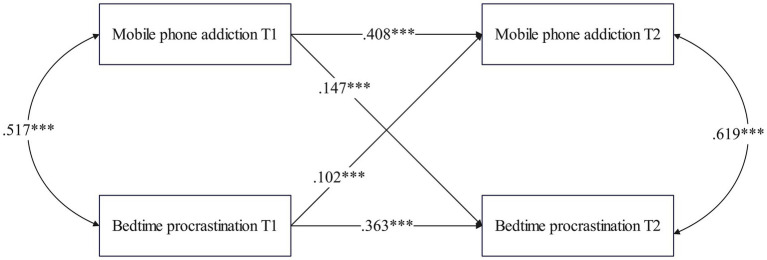
Cross-lagged path model between MPA and BP (total sample).

Cross-lagged analyses were conducted to examine the bidirectional relationship between BP and MPA among college students (χ^2^ = 388.157, *df* = 81, *p* < 0.001; CFI = 0.997, TLI = 0.986, RMSEA = 0.044, SRMR = 0.029; Figure 2). The results showed that the autoregressive paths of MPA (*β* = 0.408, SE = 0.027, *p* < 0.001) and BP (*β* = 0.363, SE = 0.027, *p* < 0.001) were significant; MPA at T1 significantly and positively predicted BP at T2 (*β* = 0.147, SE = 0.028, *p* < 0.001), i.e., the higher the degree of MPA among college students, the subsequent more pronounced BP; BP at T1 also significantly and positively predicted MPA at T2 (*β* = 0.102, SE = 0.028, *p* < 0.001), i.e., the more severe BP behavior of college students, the higher the degree of MPA. To summarize, MPA was a predictor of BP, which was also a predictor of MPA over time (see Figure 2).

### 3.5 Group comparisons by stress level in cross-lagged models

**Figure 3:**
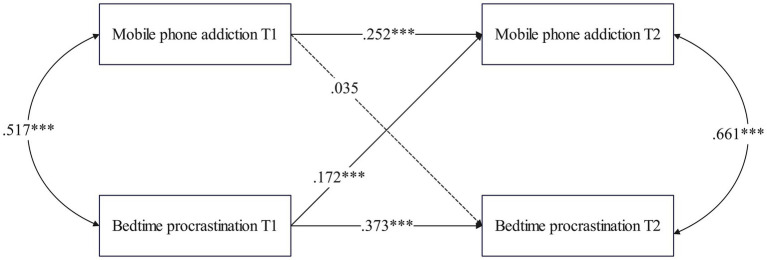
Cross-lagged path model between MPA and BP (high stress group).

The structural equation model was established to examine the cross-lagged relationship between MPA and BP in college students with low and high stress levels at T1 (χ^2^ = 387.473, *df* = 82, *p* < 0.001; CFI = 0.977, TLI = 0.962, RMSEA = 0.042, SRMR = 0.037; see Figure 3).

As illustrated in Figure 3, for the T1 high-stress group, MPA did not significantly predict BP at T2 (*β* = 0.035, *p* = 0.405). However, BP at T1 in the high-stress group significantly predicted MPA at T2 (*β* = 0.172, *p* < 0.001).

**Figure 4:**
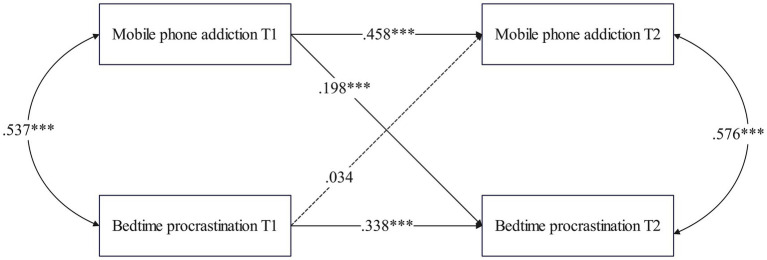
Cross-lagged path model between MPA and BP (low stress group).

Figure 4 shows that in the low-stress group, MPA at T1 significantly predicted BP at T2 (*β* = 0.198, *p* < 0.001), while BP at T1 did not significantly predict MPA at T2 (*β* = 0.034, *p* = 0.337). These findings suggest distinct cross-lagged relationships between MPA and BP in college students, with the high-stress group showing a significant reverse effect, and the low-stress group demonstrating a forward effect from MPA to BP.

### 3.6 Group comparisons by gender

**Figure 5:**
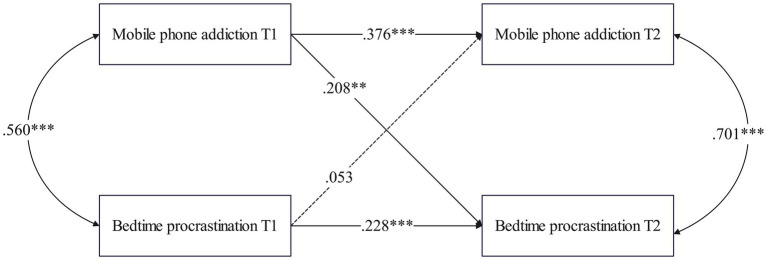
Cross-lagged path model between MPA and BP among males.

A structural equation model was used to examine the cross-lagged relationship between MPA and BP in college students of different genders (χ^2^ = 390.088, *df* = 82, p < 0.001; CFI = 0.950, TLI = 0.903, RMSEA = 0.042, SRMR = 0.067). As shown in Figure 5, for male students, MPA at T1 significantly predicted BP at T2 (*β* = 0.208, *p* = 0.002), but BP at T1 did not significantly predict MPA at T2 (*β* = 0.053, *p* = 0.422).

**Figure 6:**
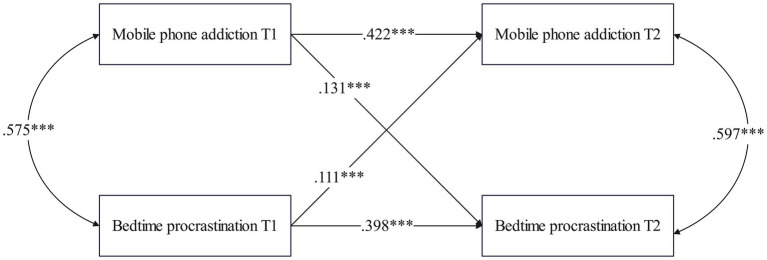
Cross-lagged path model between MPA and BP among females.

Figure 6 shows that in the females group, MPA at T1 significantly predicted BP at T2 (*β* = 0.131, *p* < 0.001). Furthermore, BP at T1 also significantly predicted MPA at T2 (*β* = 0.111, *p* < 0.001).

## 4 Discussion

This study uncovers a dynamic interplay between MPA and BP, revealing a bidirectional relationship moderated by stress levels and gender. Our findings indicate that under low-stress conditions, MPA predicts subsequent BP, whereas high-stress reverses this, with BP predicting MPA. Gender analysis further shows that while MPA predicts BP in both male and female students, BP’s prediction of MPA is significant only among female students. These findings offer fresh perspectives on stress mechanisms and provide a theoretical foundation for targeted interventions. They underscore the need for personalized strategies tailored to students of different genders and stress levels, promoting healthy development and overall well-being.

### 4.1 The bidirectional relationship between MPA and BP among college students

This study reveals a bidirectional reinforcement cycle between MPA and BP among Chinese college students, where each behavior amplifies the other through interconnected affective, cognitive, and developmental mechanisms. Recent longitudinal evidence confirms our research, with Chen et al. (2023) demonstrating cross-lagged effects between these behaviors mediated by self-control deficits, suggesting self-regulation interventions as potential solutions. Cemei et al. (2024) revealed stress levels moderate the bidirectional relationship, amplifying behavioral reinforcement. Correa-Iriarte et al. (2023) contextualized these interactions, showing BP escalation directly correlates with degraded sleep quality, creating a vicious cycle. However, our findings demonstrate reciprocal MPA-BP dynamics but extend these observations through theoretical integration. The I-PACE model (Brand et al., 2016) clarifies how emerging adults’ developmental vulnerabilities (Schulenberg et al., 2004)—including reduced parental oversight (Arnett, 2000), heightened impulsivity (Steinberg and Morris, 2001; Baumeister, 2002), and materialistic social norms (Twenge and Kasser, 2013)—interact with affective states like academic stress to impair executive functioning. This impairment manifests as attentional bias toward phone notifications (Vollmer et al., 2017) and failed self-regulation attempts (Kroese et al., 2016; Steel and Klingsieck, 2016), creating conditions where momentary stress relief through mobile phone use inadvertently delays bedtime. Behavioral Addiction Theory (Kuss and Griffiths, 2012) further enlightens, this cycle through its neurochemical lens: dopamine-driven reward loops from social interactions and entertainment (Correa-Iriarte et al., 2023; Kundakovic and Rocks, 2022) reinforce compulsive mobile phone engagement, particularly during emotional distress (Elhai et al., 2020). Concurrently, Self-Determination Theory (Ryan and Deci, 2000) reveals how unmet psychological needs drive compensatory behaviors—students overusing mobile phones to fulfill autonomy and relatedness deficits ironically disrupt sleep routines, perpetuating BP. These theoretical perspectives collectively explain why transitional life stages heightens MPA-BP comorbidity, as students navigate conflicting desires for independence and social connection against academic pressures.

Based on the dynamic characteristics of the bidirectional relationship, intervention designs should focus on integrating self-regulation training (e.g., executive function enhancement) and Cognitive Behavior Therapy (CBT)-oriented stress management modules (Simón-Grábalos et al., 2025; Nakao et al., 2021). This approach must be tightly coordinated with reforms to university curriculum systems—for instance, incorporating mindfulness training into freshman orientation programs and adding a “Digital Wellness Management” module to the mental health curriculum (Black et al., 2015; Jafar et al., 2015).

### 4.2 Group differences by perceived stress levels

Under different stress conditions, the cross-lagged relationship between MPA and BP among college students varies significantly. In high-stress environments, MPA at T1 does not significantly predict BP at T2. Among those with low stress levels, MPA at T1 significantly and positively predicts BP at T2.

As recent studies have shown that stress can lead to self-regulation failures and maladaptive coping strategies such as using mobile phones to relieve negative emotions (Kadzikowska-Wrzosek, 2020; Song and Park, 2019). Individuals under high stress are more tend to self-regulation failures, which can manifest as BP behaviors (Kroese et al., 2014). BP at T1 does not predict MPA at T2 in college students with low-stress levels because these students can effectively manage their bedtime routines and phone use, thus lightening the risk of developing MPA (Bernecker and Job, 2020). These students are better equipped to manage their phone use and maintain healthy sleep routines, which in turn reduces the likelihood of MPA.

The observed differences under varying stress conditions can be interpreted through two theoretical lenses: the Stress-Vulnerability Model (Kroese et al., 2014) and Self-Regulation Theory (Muraven and Baumeister, 2000). As suggested by Kroese et al. (2014), individuals under high stress are more prone to self-regulation failures, which can manifest as BP behaviors. College students experiencing high stress may encounter increasingly difficult events, leading to a decline in self-regulatory ability and increased emotional arousal (Kadzikowska-Wrzosek, 2020). In high-stress environments, this vulnerability dominates, leading individuals to engage in BP regardless of their baseline MPA levels. They use mobile phones or other devices as a coping mechanism to relieve negative emotions, thereby delaying bedtime (Song and Park, 2019). Furthermore, high stress can trigger rumination, involving fixating on negative content and worsening mood (Diamond, 2013; Kadzikowska-Wrzosek, 2020; Ganor et al., 2023). The overwhelming impact of stress and rumination might mask the direct influence of MPA on BP in high-stress scenarios. In addition, Muraven’s self-regulation theory suggests that individuals with lower stress levels consume fewer self-regulatory resources, allowing them to better inhibit BP and excessive phone use (Muraven and Baumeister, 2000). In low-stress environments, individuals are better able to exert control over their mobile phone use and bedtime routines. Under high stress, individuals may rely more on mobile phones for emotional regulation, seeking immediate pleasure and distraction (Csikszentmihalyi and Lefevre, 1989; Luo et al., 2017; Zhou et al., 2022). This reliance on mobile phones for emotional relief can lead to excessive use, reinforcing both MPA and BP.

Interestingly, the stress-vulnerability hypothesis (Bernecker and Job, 2020) posits that in the absence of significant stressors, positive traits such as resilience and effective coping strategies help individuals resist negative emotions and behaviors. This may explain why BP at T1 does not predict MPA at T2 in college students with low stress levels. These students can effectively manage their bedtime routines and phone use, thus mitigating the risk of developing MPA.

To help college students manage stress and mitigate the effects on MPA and BP, universities should implement integrated strategies that include time management, study skills development, and the cultivation of supportive social networks (Chen et al., 2025). At the same time, it promotes the establishment of good interpersonal relationships, provides emotional support, and further reduces stress (Alborzkouh et al., 2015).

### 4.3 Group differences by gender

In this study, group differences by gender were found. For both sexes, MPA at T1 significantly predicted BP at T2. The difference is that T1 BP significantly predicted T2 MPA in female groups, but not in male groups. Recent studies have provided support for these mechanisms. For instance, Meng et al. (2024) found that female college students tend to exhibit higher levels of MPA and BP than their male counterparts, with perceived stress and coping strategies acting as mediators. Similarly, Cui et al. (2021) reported that female students were more likely to engage in MPA and BP as a way to cope with academic and social stressors. Furthermore, Chen and Zhu (2024) discovered that rumination partially mediates the relationship between MPA and BP, while gender moderates this relationship.

To gain a clearer understanding of this pattern, we can benefit from exploring the triad of biological, sociocultural, and psychological factors underlying gender differences. From a neuroendocrine perspective, women’s heightened sensitivity to estrogen/progesterone fluctuations (Altemus et al., 2014) amplifies emotional dysregulation (Kundakovic and Rocks, 2022), frequently triggering compensatory mobile phone use for mood modulation that inadvertently elevates combined risks of MPA and BP (Graves et al., 2021). Culturally, traditional gender expectations demanding constant social availability (Zhang et al., 2018; Zhang and Wang, 2023) reinforce nighttime mobile phone engagement patterns that systematically erode sleep opportunities, thereby perpetuating BP. Psychologically, women’s predominant reliance on emotion-focused coping strategies—particularly the ruminative processing of distress proposed in Response Styles Theory (Nolen-Hoeksema et al., 2008)—creates dual vulnerability pathways: prolonged negative affect drives excessive reassurance-seeking through mobile phones (Elhai et al., 2020), while BP attempts to escape ruminative thoughts further disrupt circadian rhythms (Yang J. et al., 2020). Men tend to use problem-focused strategies, such as gaming, which lead to different ways of using technology. These differences reduce the positive effects seen in the interaction between MPA and BP, helping to explain the gender differences in these dynamics (Yang J. et al., 2020).

Thus, when designing interventions to reduce MPA and BP among college students, it is essential to consider gender differences. Campus interventions should adopt gender-differentiated approaches: for females, implement mindfulness training targeting emotional regulation and social norm reconstruction workshops to challenge connectivity expectations (Kang et al., 2018); for males, develop structured gaming alternatives with circadian-friendly schedules. Establish peer-led tech-wellness groups and curriculum-embedded modules to enhance self-regulation capacities, prioritizing concurrent MPA-BP intervention through stress-coping skill development (Ortega-Ruipérez and Correa-Gorospe, 2024).

### 4.4 Implications and limitations

The longitudinal tracking design was adopted in this study, which broke through the limitation of cross-sectional study and deeply explored the dynamic relationship between MPA and BP of college students. By establishing a cross-lagged model, the applicability and invariance of different stress levels and gender groups were analyzed. The results show that there is a bidirectional predictive relationship between MPA and BP, and stress and gender play a moderating role in this relationship. This finding not only deepens our theoretical understanding of MPA and BP but also provides a scientific basis for developing personalized intervention strategies. Based on the findings, universities should implement targeted interventions that take gender differences and stress levels into account to help students manage MPA and BP. For example, developing programs focused on emotional regulation and coping strategies for female students is crucial. Furthermore, universities should establish comprehensive wellness programs that integrate stress management, self-regulation training, and digital wellness education to help students effectively balance their academic and social lives. Creating supportive social networks is also essential; encouraging students to participate in peer-led groups focused on tech wellness can provide community support, reduce feelings of isolation, and promote healthier habits.

Although this study has achieved important results in theory and practice, there are still some limitations. First, the research sample is only from a university in Wenzhou, which lacks the representation of college students from different regions, which limits the generalization of the conclusion. Future studies should expand the sample scope to include college students from different geographical and cultural backgrounds to enhance the external validity of the study. Secondly, the sample size of this study is relatively small, especially the small number of juniors participating, which affects the representation of students in different grades. Future studies should increase the sample size of students at all grades to improve the generality of conclusions. In addition, this study did not include an intervention design, and future studies could be combined with intervention studies to better evaluate the effect of reducing MPA on reducing BP. Through intervention research, research hypotheses can be verified more directly and scientific basis for practical intervention can be provided. Self-reported questionnaires were used in this study, and there may be measurement biases. Future studies should integrate objective indicators, such as cell phone usage records and sleep monitoring devices, to improve the validity and reliability of the study. Finally, although this study explored the moderating effect of different stress levels and gender, future studies should further distinguish more demographic variables and other influencing factors to explore the applicability and invariance of the cross-lagged model of MPA and BP in more groups. Through these improvements, future studies can more comprehensively and deeply understand the relationship between MPA and BP in college students and provide a solid scientific basis for the development of more effective intervention measures.

## 5 Conclusion

This study investigated the bidirectional relationship between MPA and BP among college students using a longitudinal design, uncovering the dynamic interplay between these two constructs. The results indicated that MPA and BP were mutually predictive, with stress levels and gender moderation of this relationship. Specifically, in the low-stress group, BP predicted subsequent MPA, whereas in the high-stress group, MPA predicted subsequent BP. Additionally, gender analysis revealed that MPA significantly predicted BP in males, while a reciprocal relationship was observed in females. These findings not only enhance our understanding of the relationship between MPA and BP but also lay the groundwork for personalized intervention strategies. Future research should further validate these results and explore targeted interventions to mitigate the adverse impacts of MPA and BP on the mental and physical health of college students. Overall, this study offers a novel perspective for addressing MPA and BP among college students.

## Data Availability

The raw data supporting the conclusions of this article will be made available by the authors, without undue reservation.

## References

[ref1] Alborzkouh P. Nabati M. Zainali M. Abed Y. Shahgholy Ghahfarokhi F. (2015). A review of the effectiveness of stress management skills training on academic vitality and psychological well-being of college students. J. Med. Life 8, 39–44, PMID: 28316704 PMC5319270

[ref2] Altemus M. Sarvaiya N. Neill Epperson C. (2014). Sex differences in anxiety and depression clinical perspectives. Front. Neuroendocrinol. 35, 320–330. doi: 10.1016/j.yfrne.2014.05.004, PMID: 24887405 PMC4890708

[ref3] Arnett J. J. (2000). Emerging adulthood: a theory of development from the late teens through the twenties. Am. Psychol. 55, 469–480. doi: 10.1037/0003-066X.55.5.469, PMID: 10842426

[ref4] Baumeister R. F. (2002). Yielding to temptation: self-control failure, impulsive purchasing, and consumer behavior. J. Consum. Res. 28, 670–676. doi: 10.1086/338209

[ref5] Bernecker K. Job V. (2020). Too exhausted to go to bed: implicit theories about willpower and stress predict bedtime procrastination. Br. J. Psychol. 111, 126–147. doi: 10.1111/bjop.12382, PMID: 30854630

[ref6] Berset M. Elfering A. Lüthy S. Lüthi S. Semmer N. K. (2011). Work stressors and impaired sleep: rumination as a mediator. Stress. Health 27, e71–e82. doi: 10.1002/smi.1337, PMID: 27486625

[ref7] Black D. S. O’Reilly G. A. Olmstead R. Breen E. C. Irwin M. R. (2015). Mindfulness meditation and improvement in sleep quality and daytime impairment among older adults with sleep disturbances: a randomized clinical trial. JAMA Intern. Med. 175, 494–501. doi: 10.1001/jamainternmed.2014.8081, PMID: 25686304 PMC4407465

[ref8] Bozkurt A. Demirdöğen E. Y. Akıncı M. A. (2024). The association between bedtime procrastination, sleep quality, and problematic smartphone use in adolescents: a mediation analysis. Eurasian J. Med. 56, 69–75. doi: 10.5152/eurasianjmed.2024.23379, PMID: 39128061 PMC11059092

[ref9] Brand M. Wegmann E. Stark R. Müller A. Wölfling K. Robbins T. W.. (2019). The interaction of person-affect-cognition-execution (I-PACE) model for addictive behaviors: update, generalization to addictive behaviors beyond internet-use disorders, and specification of the process character of addictive behaviors. Neurosci. Biobehav. Rev. 104, 1–10. doi: 10.1016/j.neubiorev.2019.06.032, PMID: 31247240

[ref10] Brand M. Young K. S. Laier C. Wölfling K. Potenza M. N. (2016). Integrating psychological and neurobiological considerations regarding the development and maintenance of specific internet-use disorders: An interaction of person-affect-cognition-execution (I-PACE) model. Neurosci. Biobehav. Rev. 71, 252–266. doi: 10.1016/j.neubiorev.2016.08.033, PMID: 27590829

[ref11] Cemei L. Sriram S. Holý O. Rehman S. (2024). A longitudinal investigation on the reciprocal relationship of problematic smartphone use with bedtime procrastination, sleep quality, and mental health among university students. Psychol. Res. Behav. Manag. 17, 3355–3367. doi: 10.2147/PRBM.S472299, PMID: 39359420 PMC11446206

[ref12] Chen S. Liao J. Wang X. Wei M. Liu Y. (2023). Bidirectional relations between problematic smartphone use and bedtime procrastination among Chinese university students: self-control as a mediator. Sleep Med. 112, 53–62. doi: 10.1016/j.sleep.2023.09.033, PMID: 37806036

[ref13] Chen W. G. Zhu F. S. (2024). The relationship between mobile phone addiction and sleep procrastination among college students: a moderated mediation model. Sichuan Sports Sci. 3, 54–60. doi: 10.13932/j.cnki.sctykx.2024.03.10

[ref14] Chen C. Zhu Y. Sun Y. Que M. (2025). The relationship between social support and interpersonal self-efficacy among higher vocational college students: parallel mediation effects of anxiety and loneliness. BMC Psychol. 13:102. doi: 10.1186/s40359-025-02418-4, PMID: 39910620 PMC11796119

[ref15] China Internet Network Information Center (2024). China internet development report. Beijing, China: CNNIC.

[ref16] Chung S. J. An H. Suh S. (2020). What do people do before going to bed? A study of bedtime procrastination using time use surveys. Sleep 43:zsz267. doi: 10.1093/sleep/zsz267, PMID: 31680171

[ref17] Correa-Iriarte S. Hidalgo-Fuentes S. Martí-Vilar M. (2023). Relationship between problematic smartphone use, sleep quality, and bedtime procrastination: a mediation analysis. Behav. Sci. 13:839. doi: 10.3390/bs13100839, PMID: 37887489 PMC10604906

[ref18] Csikszentmihalyi M. Lefevre J. (1989). Optimal experience in work and leisure. J. Pers. Soc. Psychol. 56, 815–822. doi: 10.1037/0022-3514.56.5.815 2724069

[ref19] Cui G. Yin Y. Li S. Chen S. Gao X. (2021). Longitudinal relationships among problematic mobile phone use, bedtime procrastination, sleep quality, and depressive symptoms in Chinese college students: a cross-lagged panel analysis. BMC Psychiatry 21:449. doi: 10.1186/s12888-021-03451-4, PMID: 34507561 PMC8431882

[ref20] Diamond A. (2013). Executive functions. Annu. Rev. Psychol. 64, 135–168. doi: 10.1146/annurev-psych-113011-143750, PMID: 23020641 PMC4084861

[ref21] Elhai J. D. Yang H. Fang J. Bai X. Hall B. J. (2020). Depression and anxiety symptoms are related to problematic smartphone use severity in Chinese young adults: fear of missing out as a mediator. Addict. Behav. 101:105962. doi: 10.1016/j.addbeh.2019.04.020, PMID: 31030950

[ref300] Enez Darcin A. Kose S. Noyan C. O. Nurmedov S. Yılmaz O. Dilbaz N. (2016). Smartphone addiction and its relationship with social anxiety and loneliness. Behav. Inf. Technol 35, 520–525. doi: 10.1080/0144929X.2016.1158319, PMID: 32717969

[ref22] Ganor T. Mor N. Huppert J. D. (2023). Effects of rumination and distraction on inhibition. J. Behav. Ther. Exp. Psychiatry 78:101780. doi: 10.1016/j.jbtep.2022.101780, PMID: 36206674

[ref23] Gao Q. Zheng H. Sun R. Lu S. (2022). Parent-adolescent relationships, peer relationships, and adolescent mobile phone addiction: the mediating role of psychological needs satisfaction. Addict. Behav. 129:107260. doi: 10.1016/j.addbeh.2022.107260, PMID: 35151093

[ref24] Geng Y. Gu J. Wang J. Zhang R. (2021). Smartphone addiction and depression, anxiety: the role of bedtime procrastination and self-control. J. Affect. Disord. 293, 415–421. doi: 10.1016/j.jad.2021.06.062, PMID: 34246950

[ref25] Geng J. Han L. Gao F. Jou M. Huang C. (2018). Internet addiction and procrastination among Chinese young adults: a moderated mediation model. Comput. Hum. Behav. 84, 320–333. doi: 10.1016/j.chb.2018.03.013

[ref26] Gong X. Xie X. Y. Xu R. Luo Y. J. (2010). Validation of the Chinese version of the depression–anxiety–stress Scale-21 (DASS-21) among Chinese college students. Chin. J. Clin. Psych. 18, 443–446. doi: 10.16128/j.cnki.1005-3611.2010.04.020

[ref27] Graves B. S. Hall M. E. Dias-Karch C. Haischer M. H. Apter C. (2021). Gender differences in perceived stress and coping among college students. PLoS One 16:e0255634. doi: 10.1371/journal.pone.0255634, PMID: 34383790 PMC8360537

[ref28] Hamvai C. Kiss H. Vörös H. Pribék I. K. Lőrincz L. (2023). Association between impulsivity and cognitive capacity decrease is mediated by smartphone addiction, academic procrastination, bedtime procrastination, sleep insufficiency, and daytime fatigue among medical students: a path analysis. BMC Med. Educ. 23:537. doi: 10.1186/s12909-023-04522-8, PMID: 37501113 PMC10375684

[ref29] Hong W. Liu R. D. Ding Y. Shen W. Zheng Q. (2021). Academic procrastination precedes problematic mobile phone use in Chinese adolescents: a longitudinal mediation model of distraction cognitions. Addict. Behav. 121:106993. doi: 10.1016/j.addbeh.2021.106993, PMID: 34098430

[ref30] Hong W. Liu R.-D. Ding Y. Zhen R. Jiang R. Fu X. (2020). Autonomy need dissatisfaction in daily life and problematic Mobile phone use: the mediating roles of boredom proneness and Mobile phone gaming. Int. J. Environ. Res. Public Health 17:5305. doi: 10.3390/ijerph17155305, PMID: 32717969 PMC7432443

[ref31] Jafar H. M. Salabifard S. Mousavi S. M. Sobhani Z. (2015). The effectiveness of group training of CBT-based stress management on anxiety, psychological hardiness and general self-efficacy among university students. Global J. Health Sci. 8, 47–54. doi: 10.5539/gjhs.v8n6p47, PMID: 26755483 PMC4954877

[ref32] Kadzikowska-Wrzosek R. (2020). Insufficient sleep among adolescents: the role of bedtime procrastination, chronotype, and autonomous vs. controlled motivational regulations. Curr. Psychol. 39, 1031–1040. doi: 10.1007/s12144-018-9825-7

[ref33] Kang Y. Rahrig H. Eichel K. Niles H. F. Rocha T. Lepp N. E.. (2018). Gender differences in response to a school-based mindfulness training intervention for early adolescents. J. Sch. Psychol. 68, 163–176. doi: 10.1016/j.jsp.2018.03.004, PMID: 29861026 PMC6174072

[ref34] Kroese F. M. De Ridder D. T. Evers C. Adriaanse M. A. (2014). Bedtime procrastination: introducing a new area of procrastination. Front. Psychol. 5:611. doi: 10.3389/fpsyg.2014.00611, PMID: 24994989 PMC4062817

[ref35] Kroese F. M. Evers C. Adriaanse M. A. de Ridder D. T. (2016). Bedtime procrastination: a self-regulation perspective on sleep insufficiency in the general population. J. Health Psychol. 21, 853–862. doi: 10.1177/1359105314540014, PMID: 24997168

[ref36] Kundakovic M. Rocks D. (2022). Sex hormone fluctuation and increased female risk for depression and anxiety disorders: from clinical evidence to molecular mechanisms. Front. Neuroendocrinol. 66:101010. doi: 10.1016/j.yfrne.2022.101010, PMID: 35716803 PMC9715398

[ref37] Kuss D. J. Griffiths M. D. (2012). Internet gaming addiction: a systematic review of empirical research. Int. J. Ment. Heal. Addict. 10, 278–296. doi: 10.1007/s11469-011-9318-5

[ref38] Leung L. (2008). Linking psychological attributes to addiction and improper use of the mobile phone among adolescents in Hong Kong. J. Child. Media 2, 93–113. doi: 10.1080/17482790802078565

[ref39] Li Y. Li G. Liu L. Wu H. (2020). Correlations between mobile phone addiction and anxiety, depression, impulsivity, and poor sleep quality among college students: a systematic review and meta-analysis. J. Behav. Addict. 9, 551–571. doi: 10.1556/2006.2020.00057, PMID: 32903205 PMC8943681

[ref40] Li Y. Zhang X. Lu F. Zhang Q. Wang Y. (2014). Internet addiction among elementary and middle school students in China: a nationally representative sample study. Cyberpsychol. Behav. Soc. Netw. 17, 111–116. doi: 10.1089/cyber.2012.0482, PMID: 23971432 PMC3924822

[ref42] Lovibond S. H. Lovibond P. F. (1995). Manual for the depression anxiety stress scales (2nd ed.). Psychology Foundation of Australia.

[ref43] Luo Y. J. Kong F. C. Niu G. F. Fan C. Y. Zhou Z. K. (2017). The impact of stressful events on depression in junior high school students: the role of internet use motivation and intensity. Psychol. Dev. Educ. 33, 337–344. doi: 10.16187/j.cnki.issn1001-4918.2017.03.11

[ref44] Ma X. Meng D. Zhu L. Wang J. Li X. (2022). Bedtime procrastination predicts the prevalence and severity of poor sleep quality of Chinese undergraduate students. J. Am. Coll. Heal. 70, 1104–1111. doi: 10.1080/07448481.2020.1785474, PMID: 32669056

[ref45] Meng S. Zhang Y. Tang L. Sun Y. Liu X. (2024). The effects of mobile phone addiction on bedtime procrastination in university students: the masking effect of physical activity and anxiety. BMC Psychol. 12:395. doi: 10.1186/s40359-024-01899-z, PMID: 39020420 PMC11253395

[ref46] Muraven M. Baumeister R. F. (2000). Self-regulation and depletion of limited resources: does self-control resemble a muscle? Psychol. Bull. 126, 247–259. doi: 10.1037/0033-2909.126.2.247, PMID: 10748642

[ref47] Nakao M. Shirotsuki K. Sugaya N. (2021). Cognitive-behavioral therapy for management of mental health and stress-related disorders: recent advances in techniques and technologies. BioPsychoSocial Med. 15:16. doi: 10.1186/s13030-021-00219-w, PMID: 34602086 PMC8489050

[ref48] Nolen-Hoeksema S. Wisco B. E. Lyubomirsky S. (2008). Rethinking rumination. Perspect. Psychol. Sci. 3, 400–424. doi: 10.1111/j.1745-6924.2008.00088.x, PMID: 26158958

[ref49] Ortega-Ruipérez B. Correa-Gorospe J. M. (2024). Peer assessment to promote self-regulated learning with technology in higher education: systematic review for improving course design. Front. Educ. 9:1376505. doi: 10.3389/feduc.2024.1376505

[ref390] Podsakoff P. M. MacKenzie S. B. Lee J. Y. Podsakoff N. P. (2003). Common method biases in behavioral research: A critical review of the literature and recommended remedies. J. Appl. Psychol. 88, 879–903. doi: 10.1037/0021-9010.88.5.879, PMID: 14516251

[ref50] Ryan R. M. Deci E. L. (2000). Self-determination theory and the facilitation of intrinsic motivation, social development, and well-being. Am. Psychol. 55, 68–78. doi: 10.1037/0003-066X.55.1.68, PMID: 11392867

[ref500] Samaha M. Hawi N. S. (2016). Relationships among smartphone addiction, stress, academic performance, and satisfaction with life. Comput. Hum. Behav. 57, 321–325. doi: 10.1016/j.chb.2015.12.045, PMID: 11392867

[ref51] Schmidt L. I. Baetzner A. S. Dreisbusch M. I. Mertens A. Sieverding M. (2024). Postponing sleep after a stressful day: patterns of stress, bedtime procrastination, and sleep outcomes in a daily diary approach. Stress. Health 40:e3330. doi: 10.1002/smi.3330, PMID: 37846558

[ref52] Schulenberg J. E. Sameroff A. J. Cicchetti D. (2004). The transition to adulthood as a critical juncture in the course of psychopathology and mental health. Dev. Psychopathol. 16, 797–806. doi: 10.1017/S0954579404040015, PMID: 15704815

[ref53] Simón-Grábalos D. Fonseca D. Aláez M. Romero-Yesa S. Fresneda-Portillo C. (2025). Systematic review of the literature on interventions to improve self-regulation of learning in first-year university students. Educ. Sci. 15:372. doi: 10.3390/educsci15030372

[ref54] Song W. J. Park J. W. (2019). The influence of stress on internet addiction: mediating effects of self-control and mindfulness. Int. J. Ment. Heal. Addict. 17, 1063–1075. doi: 10.1007/s11469-019-0051-9

[ref55] Steel P. Klingsieck K. B. (2016). Academic procrastination: psychological antecedents revisited. Aust. Psychol. 51, 36–46. doi: 10.1111/ap.12173

[ref56] Steinberg L. Morris A. S. (2001). Adolescent development. Annu. Rev. Psychol. 52, 83–110. doi: 10.1146/annurev.psych.52.1.83, PMID: 11148300

[ref57] Twenge J. M. Kasser T. (2013). Generational changes in materialism and work centrality, 1976–2007: associations with temporal changes in societal insecurity and materialistic role modeling. Personal. Soc. Psychol. Bull. 39, 883–897. doi: 10.1177/0146167213484586, PMID: 23637277

[ref58] Van Deursen A. J. A. M. Bolle C. L. Hegner S. M. Kommers P. A. M. (2015). Modeling habitual and addictive smartphone behavior. Comput. Hum. Behav. 45, 411–420. doi: 10.1016/j.chb.2014.12.039

[ref59] Vollmer C. Jankowski K. S. Díaz-Morales J. F. Itzek-Greulich H. Randler C. (2017). Morningness-eveningness correlates with sleep time, quality, and hygiene in secondary school students: a multilevel analysis. Sleep Med. 30, 151–159. doi: 10.1016/j.sleep.2016.09.022, PMID: 28215240

[ref60] Winkler A. Jeromin F. Doering B. K. Barke A. (2020). Problematic smartphone use has detrimental effects on mental health and somatic symptoms in a heterogeneous sample of German adults. Comput. Hum. Behav. 113:106500. doi: 10.1016/j.chb.2020.106500

[ref61] Xu Y. J. (2017). Conceptual exploration and mechanism study of bedtime procrastination among college students (Master’s thesis. Zhejiang Normal University). Master.

[ref62] Yang J. Fu X. Liao X. Li Y. (2020). Association of problematic smartphone use with poor sleep quality, depression, and anxiety: a systematic review and meta-analysis. Psychiatry Res. 284:112686. doi: 10.1016/j.psychres.2019.112686, PMID: 31757638

[ref63] Yang X. Wang P. Hu P. (2020). Trait procrastination and mobile phone addiction among Chinese college students: a moderated mediation model of stress and gender. Front. Psychol. 11:614660. doi: 10.3389/fpsyg.2020.614660, PMID: 33335504 PMC7735984

[ref64] Yusuf A. Rachmawati P. D. Rachmawati D. (2020). The correlation of internet addiction towards adolescents’ social interaction. Int. J. Adolesc. Med. Health 34, 351–355. doi: 10.1515/ijamh-2020-0110, PMID: 32833664

[ref65] Zeng H. M. (2019). Psychological mechanisms of short video addiction: a case study of Douyin. New Media Res. 20, 16–17. doi: 10.16604/j.cnki.issn2096-0360.2019.20.004

[ref66] Zhang W. Wang Z. H. (2023). The relationship between negative life events and internalizing problems among adolescents: the mediating role of social support and the moderating role of parent-child cohesion. Psychol. Dev. Educ. 39, 718–725. doi: 10.16187/j.cnki.issn1001-4918.2023.05.13

[ref67] Zhang M. Xiao T. Zhu L. Y. (2019). Research progress on the antecedents, consequences, and interventions of mobile phone dependence. Chin. J. Spec. Educ. 11, 88–96.

[ref68] Zhang M. Zhang J. Zhang F. Zhang L. Feng D. (2018). Prevalence of psychological distress and the effects of resilience and perceived social support among Chinese college students: does gender make a difference? Psychiatry Res. 267, 409–413. doi: 10.1016/j.psychres.2018.06.038, PMID: 29960938

[ref69] Zhou H. L. Zhang B. Jiang H. B. Wang J. X. Li D. (2022). The influence of negative life events on mobile phone dependence: the mediating roles of perceived social support and relative deprivation. Chin. J. Health Psychol. 30, 971–975. doi: 10.13342/j.cnki.cjhp.2022.07.003

[ref450] Zhu Q. Gong J. Liu C. Liu Z. Yuan Y. Sun M. (2009). A study on the relationship between text message interaction behavior and anxiety among 513 college students in Jiangsu Province. Chin. J. Health Psychol. 17, 319–322. doi: 10.13342/j.cnki.cjhp.2009.03.037, PMID: 39020420

